# Signature of quantum interference effect in inter-layer Coulomb drag in graphene-based electronic double-layer systems

**DOI:** 10.1038/s41467-023-37197-2

**Published:** 2023-03-16

**Authors:** Lijun Zhu, Xiaoqiang Liu, Lin Li, Xinyi Wan, Ran Tao, Zhongniu Xie, Ji Feng, Changgan Zeng

**Affiliations:** 1 CAS Key Laboratory of Strongly-Coupled Quantum Matter Physics, and Department of Physics, University of Science and Technology of China, Hefei, 230026 China; 2 International Center for Quantum Design of Functional Materials (ICQD), Hefei National Research Center for Physical Sciences at the Microscale, University of Science and Technology of China, Hefei, 230026 China; 3 International Center for Quantum Materials, School of Physics, Peking University, Beijing, 100871 China; 4 Hefei National Laboratory, Hefei, 230088 China

**Keywords:** Electronic properties and materials, Quantum physics, Two-dimensional materials

## Abstract

The distinguishing feature of a quantum system is interference arising from the wave mechanical nature of particles which is clearly central to macroscopic electronic properties. Here, we report the signature of quantum interference effect in inter-layer transport process. Via systematic magneto-drag experiments on graphene-based electronic double-layer systems, we observe low-field correction to the Coulomb-scattering-dominated inter-layer drag resistance in a wide range of temperature and carrier density, with its characteristics sensitive to the band topology of graphene layers. These observations can be attributed to a new type of quantum interference between drag processes, with the interference pathway comprising different carrier diffusion paths in the two constituent conductors. The emergence of such effect relies on the formation of superimposing planar diffusion paths, among which the impurity potentials from intermediate insulating spacer play an essential role. Our findings establish an ideal platform where the interplay between quantum interference and many-body interaction is essential.

## Abstract (web summary)

Previous demonstrations of quantum interference in solids have mainly been limited to intra-layer transport within single conductors. Zhu et al. report a new type of inter-layer quantum interference in graphene-based double-layer devices, due to interference between carrier diffusion paths across the constituent layers.

## Introduction

As a peculiarity of quantum mechanics, quantum interference (QI) occurs between two pathways that connect the same initial and final states, demonstrating the wave-particle duality of matter and leading to many intriguing phenomena. Ever since Young’s double slit experiment in 1801^1^, exploration of QI effect has remained a topic of great interest across multiple research fields, including optics^2–4^, atomic and molecular physics^5^, solid state physics^6^, chemistry^7^, etc. One important direction is to explore the wave nature of various quantum mechanical entities using QI, with particles ranging from photons^2^ and electrons^8,9^ to heavy molecules^10,11^. Another active area of study involves detecting and manipulating possible QI occurring between more sophisticated pathways. Representative examples include the interference between two reaction or collision pathways^12–14^, which is essential to the quantum control of chemical reactions.

For solids, the quantum transport properties are strongly related to the wave-like character of electrons, and QI is more known as unique transport phenomena that normally emerge at low temperatures, such as weak localization^6,15^, universal conductance fluctuations^16,17^, and Aharonov–Bohm effect^18,19^. However, all these effects root in the interference between electron diffusion paths in a single conductor within a non-interacting single-particle framework. Further extending QI into more diversified transport processes remains to be explored.

Coulomb drag between two closely spaced but electrically isolated conductors, wherein moving carriers in one layer (active layer) induces the transport of carriers in another layer (passive layer), has been utilized as a versatile probe to detect multi-particle interactions^20^ and inter-layer coherent states^21,22^. Exploiting possible quantum phenomena in such a delicate inter-layer transport process will promote our capability to clarify the interaction mechanism and to uncover novel many-body effects. In this study, systematic magneto-drag experiments on graphene-based electronic double-layer systems are performed, from which striking low-field correction to the classical drag resistance is revealed. Such correction is found to be sensitive to the band topology of the constituent graphene layers, and can be attributed to the emergence of inter-layer QI between different drag processes.

## Results

**Fig. 1:**
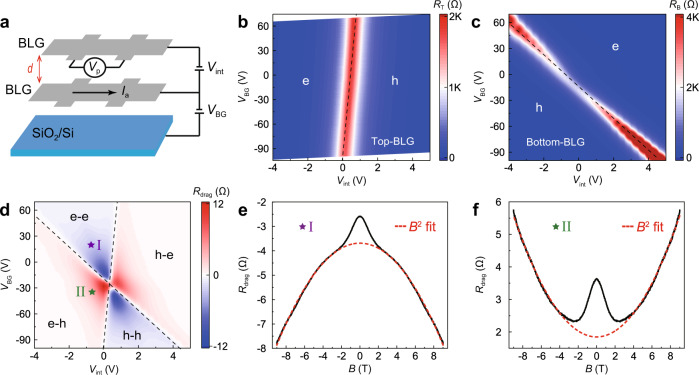
Typical drag behaviors in the bilayer/bilayer graphene (BLG/BLG) device. **a** Schematic of the drag set-up for the BLG/BLG device. The top and bottom BLG layers are separated by hBN layer with the thickness *d*~11.3 nm. **b**, **c** Resistances of top BLG layer (*R*_T_) and bottom BLG layer (*R*_B_) as functions of *V*_int_ and *V*_BG_, respectively. **d** Drag resistance (*R*_drag_) as a function of *V*_int_ and *V*_BG_. Dashed lines correspond to the charge neutrality lines of the two BLG layers, which divide the mapping into four regions. **e**, **f** Typical *R*_drag_ vs. *B* curves in the e-e region for point “I” and the e-h region for point “II” (as indicated in **d**). Red dashed curves are quadratic fits to the data. All these measurements are conducted at *T* = 200 K.

Figure 1a shows the schematic of the drag set-up utilizing a device composed of double layers of bilayer graphene (denoted as BLG/BLG device), in which a hexagonal boron nitride (hBN) layer serves as the insulating spacer. This heterostructure is further encapsulated by hBN layers and then assembled on a SiO_2_/Si substrate (see Methods for the fabrication details). By utilizing the inter-layer-gate voltage (*V*_int_) and back-gate voltage (*V*_BG_), the carrier density and polarity for each BLG layer can be readily tuned, as respectively demonstrated in Fig. 1b, c.

Drag experiments were carried out by applying current *I*_a_ to the bottom BLG layer and measuring the induced open-circuit voltage *V*_p_ in the top BLG layer. The linear dependence of *V*_p_ against *I*_a_, the negligibility of inter-layer leakage current, and the Onsager reciprocity relation were carefully checked to confirm the validity of the drag measurements (see Supplementary Note 1). Figure 1d shows the obtained drag resistance (*R*_drag_ = *V*_p_/*I*_a_) as a function of *V*_int_ and *V*_BG_ measured at 200 K. Four distinct regions can be identified from the mapping data: electron-electron (e-e), hole-hole (h-h), electron-hole (e-h) and hole-electron (h-e), with the charge neutrality lines of the two BLG layers serving as the boundaries (dashed lines). *R*_drag_ is negative (positive) when the carrier polarities of two layers are the same (opposite), obeying the typical momentum transfer mechanism^23–26^.

**Fig. 2:**
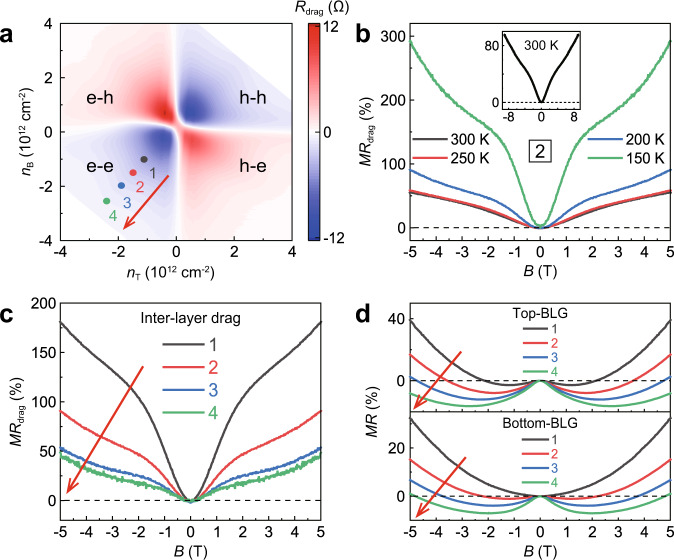
Evolution of magneto-drag behaviors with varying temperatures and carrier densities. **a** *R*_drag_ vs. (*n*_T_, *n*_B_) at *T* = 200 K, which is extracted from the data shown in Fig. 1d. **b** Magneto-drag resistance (*MR*_drag_) curves measured at different temperatures for point “2” indicated in (**a**) (the same as point “I” indicated in Fig. 1d). The 300 K result is further shown in the inset. **c** *MR*_drag_ curves for typical cases with nearly equal *n*_T_ and *n*_B_ measured at 200 K (as indicated by the dots in **a**). **d** Corresponding intra-layer *MR* curves of the top and the bottom BLG layers.

For graphene-based electronic double-layer systems, drag effects under magnetic field have been widely investigated^24,25,27–33^. However, these previous studies mainly focused on the magneto-drag performance near the charge neutrality point (CNP) of the constituent graphene layers^24,25,27,28^, or the realization of quantized Hall drag at ultra-low temperatures^29–33^. Here, we instead examine the magneto-drag behaviors away from the CNPs (carrier density >5 × 10^11^ cm^−2^) and at relatively high temperatures. Figure 1e shows a typical *R*_drag_ curve as a function of vertical magnetic field (*B*) at 200 K taken in the e-e region. An overall quadratic dependence is clearly seen within the measured range, with no signature of saturation at *B* up to 9T. Similar result has been obtained in previous drag experiments conducted at elevated temperatures^24,31,34^, which can be well depicted using the Drude-like model for Coulomb drag^27^ (as detailed in Supplementary Note 2). Strikingly, clear deviation from the *B*^2^ dependency occurs in the low-field regime (<2T), manifesting as a remarkable drag resistance peak. This observation is strongly reminiscent of the characteristic magnetoresistance (*MR*) behavior of the weak (anti-)localization effect^35–37^. The corresponding magneto-drag resistance [*MR*_drag_, defined as (*R*_drag_(*B*) − *R*_drag_(0))/*R*_drag_(0) × 100%] curves in Fig. 2b demonstrate that the low-field deviation gets suppressed with increasing temperature, also suggesting its quantum nature. For the e-h region, similar drag resistance peak is also observed in the low-field regime (Fig. 1f). However, since *R*_drag_ is positive (negative) for the e-h (e-e) region, such resistance peak manifests as the low-field negative *MR*_drag_ in the e-h region (Supplementary Fig. 4), opposite to that in the e-e region.

**Fig. 3:**
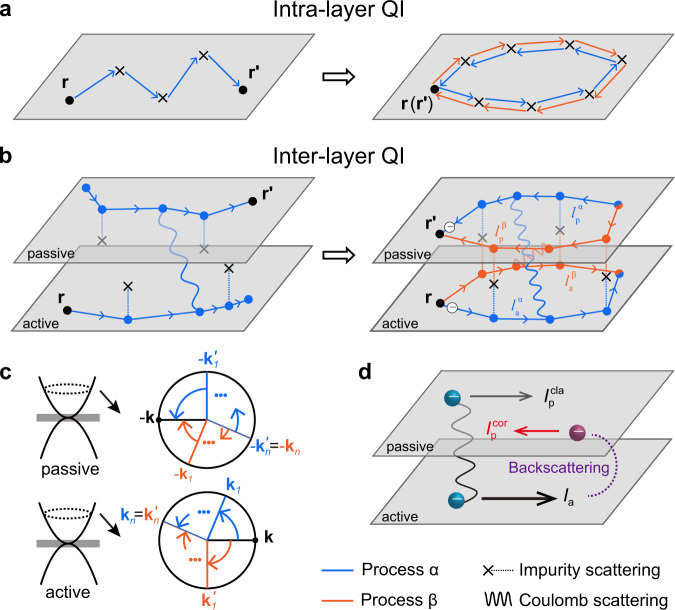
Quantum interference (QI) in Coulomb drag. **a** Intra-layer QI. Left panel: Schematic of an ordinary diffusion path from position ${{{{{\bf{r}}}}}}$ to ${{{{{{\bf{r}}}}}}}^{\prime }$. Right panel: Intra-layer QI occurring between a pair of time-reversed closed loops with ${{{{{\bf{r}}}}}}$ = ${{{{{{\bf{r}}}}}}}^{\prime}$. **b** Inter-layer QI. Left panel: Schematic of a typical drag process, which describes how an active carrier at position ${{{{{\bf{r}}}}}}$ induces a passive carrier moving toward ${{{{{{\bf{r}}}}}}}^{\prime}$. Here, only the impurity scatterings originating from the intermediate hBN spacer are shown. Right panel: Inter-layer QI between two drag processes (process “α” and “β”) in the e-e region. ${l}_{{{{{{\rm{a}}}}}}}^{{{\alpha }}/{{\beta }}}$ and ${l}_{{{{{{\rm{p}}}}}}}^{{{\alpha }}/{{\beta }}}$ are the diffusion paths of drag process ${{\alpha }}/{{\beta }}$ in the active and passive layers, respectively. ${{{{{\bf{r}}}}}}$ and ${{{{{{\bf{r}}}}}}}^{\prime}$ now have the same in-plane coordinates. **c** Reciprocal-space schematic of the inter-layer QI in the e-e region. **d** Inter-layer-QI-induced quantum correction to the drag signal in the e-e region. The classical- and inter-layer-QI-induced drag currents (*I*_p_^cla^ and *I*_p_^cor^) are indicated by the gray and red arrows, respectively.

As illustrated in Fig. 3b (left panel), Coulomb drag occurs when the carriers driven by an external electric field in the active layer induce the movement of carriers in the passive layer. Such a transport process involves carrier diffusion in the two constituent layers and inter-layer Coulomb scattering, and has been demonstrated to be tightly correlated with the inherent electronic properties of the constituent conductors^38–40^. For example, the observed giant drag fluctuations in GaAs double quantum wells were revealed to originate from the coherent electron transport within each quantum well^39^. It is therefore natural to conjecture that the low-field deviation of *MR*_drag_ observed in this study is a manifestation of weak localization within the BLG layers on the drag behaviors.

We then compared the inter-layer *MR*_drag_ and the intra-layer *MR* by investigating the evolution behaviors with varying carrier densities. Figure 2c shows the *MR*_drag_ curves in the e-e region for the cases that the carrier densities of the top and bottom BLG layers (*n*_T_ and *n*_B_) are nearly equal (as indicated in Fig. 2a). All of them possess similar features, specifically, an overall positive *MR*_drag_ with *B*^2^ dependence and a clear deviation occurring within the low-field regime. As the carrier densities increase, the low-field deviation becomes monotonically weaker. Similar trend has also been observed in other three regions (Supplementary Fig. 5), and further in another BLG/BLG device (Supplementary Fig. 6), indicating its universality. While from the *MR* curves of the two BLG layers shown in Fig. 2d, characteristic signature of weak localization is clearly observable, manifesting as a negative *MR* in the low-field regime. This effect gets significantly enhanced with increasing carrier densities, mainly due to the increase of inter-valley scattering^35,36^. The strikingly opposite carrier-density dependences for the low-field correction of inter-layer *MR*_drag_ and that of intra-layer *MR* suggest different origins for these two phenomena. This result consists with previous theoretical studies, which also suggested that weak localization within the layers has negligible contribution to the drag resistance^41,42^ (see also the mathematical analysis in Supplementary Note 5).

For the aforementioned weak localization effect, a type of intra-layer QI, interference occurs between a closed diffusion path and its time-reversed conjugate within the single layer (see Fig. 3a). More generally, interference could in principle happen when two pathways lead to identical outcome for a quantum event. This should still hold for the inter-layer drag response, wherein a more sophisticated drag process instead of a single diffusion path needs to be taken into consideration. Supposing there is a pair of drag processes *α* and *β* possessing the same initial and final states, e.g., a carrier in the active layer (“active carrier”) starting at the in-plane coordinate ${{{{{\bf{r}}}}}}$ scatters another carrier in the passive layer (“passive carrier”) to position ${{{{{{\bf{r}}}}}}}^{{\prime} }$(see the left panel of Fig. 3b), the total probability is $P\left({{{{{{\bf{r}}}}}}}^{{\prime} },{{{{{\bf{r}}}}}}\right)={\left|{\psi }_{{{\alpha }}}+{\psi }_{{{\beta }}}\right|}^{2}$, with the probability amplitude of the drag process given by $\psi$. As compared with the intra-layer QI, we need further consider the impact of inter-layer Coulomb scattering on the phase coherence of drag process. For the inter-layer Coulomb scattering, it will transfer energy $\triangle \varepsilon$ from the active layer to the passive layer, and thus introduce phase factors ${e}^{{{{{{\rm{i}}}}}}\triangle \varepsilon t}$ and ${e}^{-{{{{{\rm{i}}}}}}\triangle \varepsilon t}$ to the propagating amplitudes of the active carrier and passive carrier, respectively. However, these two phase factors will cancel out with each other eventually for the probability amplitude $\psi$ of the whole drag process (as detailed in Supplementary Note 4A). Therefore, drag processes can maintain their phase coherence and the resulting interference contribution is $2{{{{{\rm{|}}}}}}{\psi }_{{{\alpha }}}{\psi }_{{{\beta }}}{{{{{\rm{|}}}}}}{{\cos }}({\varphi }_{{{\alpha }}}-{\varphi }_{{{\beta }}})$ ($\varphi$: phase of $\psi$).

In general, interferences of nearly all pairs of drag processes cancel out after being averaged over all possible diffusion paths. Only pairs of drag processes with a constant ${\varphi }_{{{\alpha }}}-{\varphi }_{{{\beta }}}$ that are independent of constituent paths will have observable interference effect that contributes to the drag signal. This will occur when each diffusion path has a partner with a definite phase relation (either a constant phase difference or a sign reversal), which could be realized when they are interrelated by some special symmetry operations. The aforementioned intra-layer QI corresponds to the case that such two paths reside in the same layer and are interrelated via the time reversal^15^. We therefore wonder if QI could also emerge when one diffusion path and its partner reside in two separate layers. This is possible for the present electronic double-layer systems with relatively small inter-layer spacing, wherein the impurity potential originating from the intermediate insulating spacer could be equally felt by carriers in both BLG layers. Under such condition, it is possible that carriers from different BLG layers maintain their motions along superimposing planar paths^43^, which meets the above-mentioned requirement of definite phase relation. Supplementary Fig. 8 shows all the possible scenarios for interferential drag processes containing four pairs of superimposing planar paths. Below, we restrict our discussion to the case of e-e region. Similar analysis is applicable for the other three regions, as detailed in Supplementary Note 4.

After scrutinizing all possible scenarios shown in Supplementary Fig. 8 in the e-e region, we first reveal that the phase difference ${\varphi }_{{{{{{\rm{\alpha }}}}}}}-{\varphi }_{{{{{{\rm{\beta }}}}}}}$ for the interference shown in Fig. 3b (right panel) is zero, corresponding to the constructive interference (see also Supplementary Note 4). In this scenario, each pair of superimposing planar paths, i.e., ${l}_{{{{{{\rm{a}}}}}}}^{{{{{{\rm{\alpha }}}}}}}$ and ${l}_{{{{{{\rm{p}}}}}}}^{{{{{{\rm{\beta }}}}}}}$, ${l}_{{{{{{\rm{a}}}}}}}^{{{{{{\rm{\beta }}}}}}}$ and ${l}_{{{{{{\rm{p}}}}}}}^{{{\alpha }}}$, are interrelated by the inter-layer mirror reflection in addition to time reversal. The effect of such a novel interference, which we term inter-layer QI, on the drag behavior is more clearly exemplified in the reciprocal space. As schematically shown in Fig. 3c, ${l}_{{{{{{\rm{a}}}}}}}^{{{\alpha }}}$ and ${l}_{{{{{{\rm{a}}}}}}}^{{{\beta }}}$, i.e., the two diffusion paths in the active layer, can be expressed as sequences of momenta $\{{{{{{\bf{k}}}}}},{{{{{{\bf{k}}}}}}}_{1},\ldots,{{{{{{\bf{k}}}}}}}_{n}\}$ and $\{{{{{{\bf{k}}}}}},{{{{{{\bf{k}}}}}}}_{1}^{{\prime} },\ldots,{{{{{{\bf{k}}}}}}}_{n}^{{\prime} }\}$, respectively, and they form a close loop since ${{{{{{\bf{k}}}}}}}_{n}={{{{{{\bf{k}}}}}}}_{n}^{{\prime} }$. Via applying inter-layer mirror reflection and time reversal to ${l}_{{{{{{\rm{a}}}}}}}^{{{\alpha }}}$ $({l}_{{{{{{\rm{a}}}}}}}^{{{\beta }}})$, we can accordingly get ${l}_{{{{{{\rm{p}}}}}}}^{{{\beta }}}$ (${l}_{{{{{{\rm{p}}}}}}}^{{{\alpha }}}$) in the passive layer expressed as $\{-{{{{{{\bf{k}}}}}}}_{n},\ldots,{{{{{{\boldsymbol{-}}}}}}{{{{{\bf{k}}}}}}}_{1},-{{{{{\bf{k}}}}}}\}$ ($\{-{{{{{{\bf{k}}}}}}}_{n}^{{\prime} },\ldots,-{{{{{{\bf{k}}}}}}}_{1}^{{\prime} },-{{{{{\bf{k}}}}}}\}$). Therefore, the drag process involved in the inter-layer QI corresponds to the case that an active electron with momentum ${{{{{\bf{k}}}}}}$ scatters a passive electron into the ${{{{{\boldsymbol{-}}}}}}{{{{{\bf{k}}}}}}$ state. This can be referred to as an inter-layer version of backscattering between two electrons from two separate conductors in proximity. Consequently, an additional drag current (*I*_p_^cor^) emerges in the passive layer due to the enhancement of inter-layer backscattering, with its direction opposite to the drive current *I*_a_. Note that for the e-e region, the classical momentum-transfer-induced drag current (*I*_p_^cla^) flows along the same direction as *I*_a_, such that the inter-layer QI leads to a reduction in the total drag current (*I*_p_^cla^ + *I*_p_^cor^) (Fig. 3d). The corresponding decrease in accumulated open-circuit voltage between the electrodes (i.e., the measured *V*_p_) leads to a decrease in the magnitude of *R*_drag_. This is consistent with the experimentally observed deviation of zero-field *R*_drag_ (Fig. 1e). When a magnetic field is applied, the phase interference is destroyed, giving rise to a positive *MR*_drag_ in the low-field regime (Fig. 2b, c).

The newly-introduced inter-layer QI in Coulomb drag obeys the same rules of intra-layer QI. The deduced interference processes (Fig. 3b and Supplementary Fig. 9a) can be readily converted back into the Feynman diagrams (Supplementary Fig. 10) and the inter-layer Cooperon ladder emerges as expected. For such a more sophisticated transport process, the emergence of interference requires the formation of superimposing planar paths across different BLG layers, during which the nearly equal impurity potentials acting on the two layers from intermediate hBN layer plays a dominant role. Accordingly, the observed suppression of quantum correction with increasing carrier densities (Fig. 2c) may arise from the enhanced screening of intermediate impurity potentials. The persistence of quantum correction at temperatures up to 300 K (Fig. 2b) clearly benefits from the high robustness against thermal dephasing for each graphene layer in our drag devices. This is due to the fact that the inherent electron-phonon scattering in graphene is relatively weak^44,45^, while the encapsulating hBN layers and the nearby graphene layer could further screen the phonon scattering and inelastic electron-electron interaction^46–48^, thus protecting the phase coherence of diffusion paths.

On the other hand, we note that the frictional drag is inherently an inter-layer interaction effect, which should be the reason for the distinct performances of the as-emergent QI from the intra-layer one. In addition to the opposite carrier-density dependence, the magnitude of low-field correction of *MR*_drag_ is apparently higher than that of intra-layer *MR* induced by weak localization. Taking the case with relatively small (*n*_T_, *n*_B_) for example (point 1 in Fig. 2c), the *MR*_drag_ value reaches above 100% at 2T, exhibiting a strong modulation effect of the magnetic field. In comparison, the largest value of the intra-layer *MR* is below 20% at 2T (Fig. 2d). Although the high magnitude of QI-induced *MR*_drag_ require further theoretical investigations, our drag device could be a good candidate for developing new-principle magnetic memory devices.

**Fig. 4:**
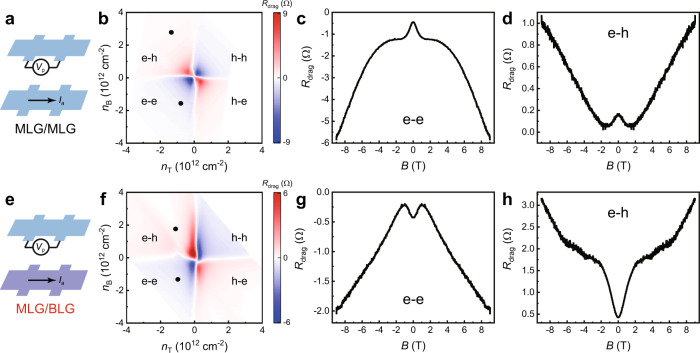
Typical magneto-drag data for the monolayer/monolayer graphene (MLG/MLG) and monolayer/bilayer graphene (MLG/BLG) devices. **a** Schematic of the MLG/MLG device and **b** the measured *R*_drag_ vs. (*n*_T_, *n*_B_) mapping. The insulating spacing of this device is ~11.7 nm. **c**, **d** *R*_drag_ vs. *B* curves for points indicated in (**b**). **e** Schematic of the MLG/BLG device and **f** the measured *R*_drag_ vs. (*n*_T_, *n*_B_) mapping. The insulating spacing is ~12 nm. **g**, **h** *R*_drag_ vs. *B* curves for points indicated in (**f**). All these measurements are conducted at *T* = 200 K.

As is well known, the geometry phase of electron wavefunction plays a key role in determining the performance of QI effect. For example, an electron in a monolayer graphene (MLG) making a round-trip on the Fermi surface acquires a nontrivial Berry phase of ${{{{{\rm{\pi }}}}}}$^49,50^, which suppresses electron backscattering and leads to weak anti-localization instead of weak-localization^36^. We next explore the effect of band topology on the magneto-drag behaviors by replacing one or two BLG layers with MLG, and the two new drag devices of MLG/MLG and MLG/BLG are schematically shown in Fig. 4a, e, respectively. Figure 4c, d shows the typical magneto-drag data for the MLG/MLG device, from which low-field deviations from the *B*^2^ dependence are clearly seen in both the e-e and e-h regions. Moreover, such deviations possess identical behaviors with those observed from the BLG/BLG device, including the peak structure, the large magnitude, as well as the carrier-density dependence (see Supplementary Fig. 11). While for the MLG/BLG device, low-field corrections are also observed (Fig. 4g, h). However, a valley feature is observed, distinctly differing from the peak feature observed in the BLG/BLG and MLG/MLG devices.

The effect of band topology can be reasonably explained using the inter-layer QI model when considering Berry phase. As shown in Fig. 3c and Supplementary Fig. 9b, the diffusion paths of the interferential drag processes form a closed loop in the reciprocal space in each layer. Consequently, carriers in the MLG and the BLG layer will acquire a Berry phase of ${{{{{\rm{\pi }}}}}}$ and 2${{{{{\rm{\pi }}}}}}$, respectively. The overall contribution of Berry phase to the phase difference ${\varphi }_{{{{{{\rm{\alpha }}}}}}}-{\varphi }_{{{{{{\rm{\beta }}}}}}}$ will be then 0 for the MLG/MLG system and ${{{{{\rm{\pi }}}}}}$ for the MLG/BLG system. That is, inter-layer QI remains constructive in the MLG/MLG devices, but turns into destructive in the MLG/BLG device, consisting well with our observation presented in Fig. 4. The band topology dependence for the observed low-fields corrections can further exclude other possible mechanisms besides the intra-layer weak localization, such as the Kondo effect^51,52^, electron-boundary scattering^53,54^ and electron-electron interaction^55,56^, etc.

In summary, the present study has revealed the signature of QI in Coulomb drag, a relatively sophisticated process consisting of carrier diffusion in two constituent layers and inter-layer Coulomb scattering. This newly-discovered inter-layer QI extends the scope of electronic interference in solids, from a non-interacting single-particle framework to an inter-layer many-body framework where Coulomb interaction plays an indispensable role. In addition, our findings provide a new approach to shape the delicate long-range interactions between quasiparticles, i.e., by controlling the distribution and strength of impurity potential via designing the intermediate dielectric spacer, which is expected to give rise to novel inter-layer quantum effects. From an engineering perspective, the strong modulation of magnetic field on drag resistance induced by inter-layer QI may have applications in new-principle electronic devices.

## Methods

### Device fabrication

BLG flakes were mechanically exfoliated from Kish graphite. The layer number and quality of the flakes were identified by combining optical microscopy and Raman spectroscopy^57,58^ (Supplementary Fig. 1a, b). Via a typical van der Waals assembly technique^59^, a heterostructure consisting of five layers of exfoliated layered materials (hBN-BLG-hBN-BLG-hBN) was then stacked on a SiO_2_/Si substrate. After shaping the heterostructure into a multi-terminal crossed electrode geometry by electron-beam lithography and reactive ion etching, one-dimensional contact electrodes (1 nm Cr/7 nm Pd/45 nm Au) were deposited on the edges of the two BLG layers by electron beam evaporation (Supplementary Fig. 1c). The device was then annealed at 350 °C in Ar/H_2_ atmosphere in order to remove chemical residues and improve contact between graphene and electrodes. The same procedure was used for the fabrication of MLG/MLG and MLG/BLG devices.

### Electronic transport measurements

Transport measurements were performed in a ^4^He cryostat (Oxford Instruments). Keithley 6220 and 2182A were used to apply currents and measure voltages, respectively. The drive current for the drag measurements was set to 1 µA based on the linear *V*_p_–*I*_a_ curves (as typically shown in Supplementary Fig. 3). The voltage background of 2182A was eliminated by applying the current in a bipolar mode, such that the final voltage was an average of measured voltages for positive and negative currents. The leakage current was quite low (<1 nA) when inter-layer-gate voltage (*V*_int_) was in the measurement range of −4 V to 5 V, ensuring the negligible contribution of inter-layer tunneling to the measured *R*_drag_ (see Supplementary Fig. 1d).

## Supplementary information

Supplementary Information

## Data Availability

The data represented in Figs. 1, 2 and 4 are available as Source Data files. All other data that support the plots within this paper and other findings of this study are available from the corresponding author on reasonable request. Source data are provided with this paper.

## Source data

Source Data
