# Supplementary material for: Signature of quantum interference effect in inter-layer Coulomb drag in graphene-based electronic double-layer systems
Source: Nat Commun. 2023 Mar 16;14:1465. doi: 10.1038/s41467-023-37197-2 (PMC10020572; doi:10.1038/s41467-023-37197-2)
Supplement: Supplementary file 1 — Supplementary Information [file 41467_2023_37197_MOESM1_ESM.pdf]

Supplementary Information for:

Signature of quantum interference effect in inter-layer Coulomb drag in graphene-based electronic double-layer systems

Lijun Zhu[1,2#], Xiaoqiang Liu[3#], Lin Li[1,2,4*], Xinyi Wan[1,2], Ran Tao[1,2], Zhongniu Xie[1,2], Ji Feng[3,4*], Changgan Zeng[1,2,4*]

[1]*CAS Key Laboratory of Strongly-Coupled Quantum Matter Physics, and Department of Physics, University of Science and Technology of China, Hefei, 230026, China*

[2]*International Center for Quantum Design of Functional Materials (ICQD), Hefei National Research Center for Physical Sciences at the Microscale, University of Science and Technology of China, Hefei, 230026, China*

[3]*International Center for Quantum Materials, School of Physics, Peking University, Beijing 100871, China*

[4]*Hefei National Laboratory, Hefei 230088, China*

[#] These authors contributed equally: Lijun Zhu, Xiaoqiang Liu.

* Correspondence and requests for materials should be addressed to C. Z. (cgzeng@ustc.edu.cn), L. L. (lilin@ustc.edu.cn) and J. F. (jfeng11@pku.edu.cn).

Supplementary Figure

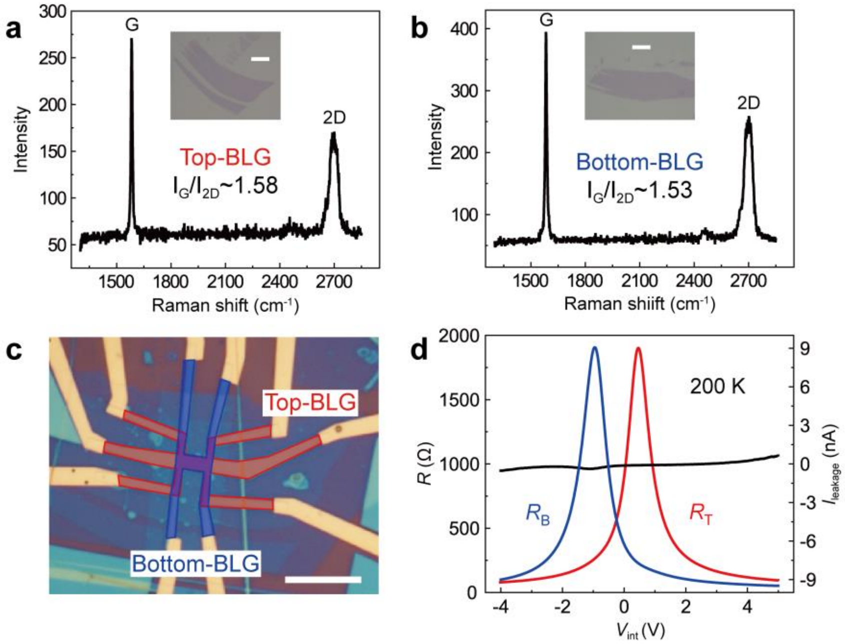

Supplementary Figure 1 | Basic characterizations of bilayer/bilayer graphene (BLG/BLG) device. Raman spectra of **a**, top-BLG and **b**, bottom-BLG after exfoliated onto SiO_2/Si substrates. Here, the G peak at 1582 cm^{-1} is associated with the doubly degenerate phonon mode at the Brillouin zone center (E_g mode), while the 2D peak around 2700 cm^{-1} is derived from the inelastic scattering of second-order zone-boundary phonons. I_G and I_{2D} represent the Raman intensity of the G peak and the 2D peak, respectively. The scale bars in the insets are 10 μm. **c**, Optical image of the final BLG/BLG device. The scale bar is 10 μm. **d**, Resistance of the top and bottom BLG layers (R_T, R_B) and inter-layer leakage current ($I_{leakage}$) as functions of V_{int} measured at 200 K. V_{BG} is set to be 0 during the measurements.

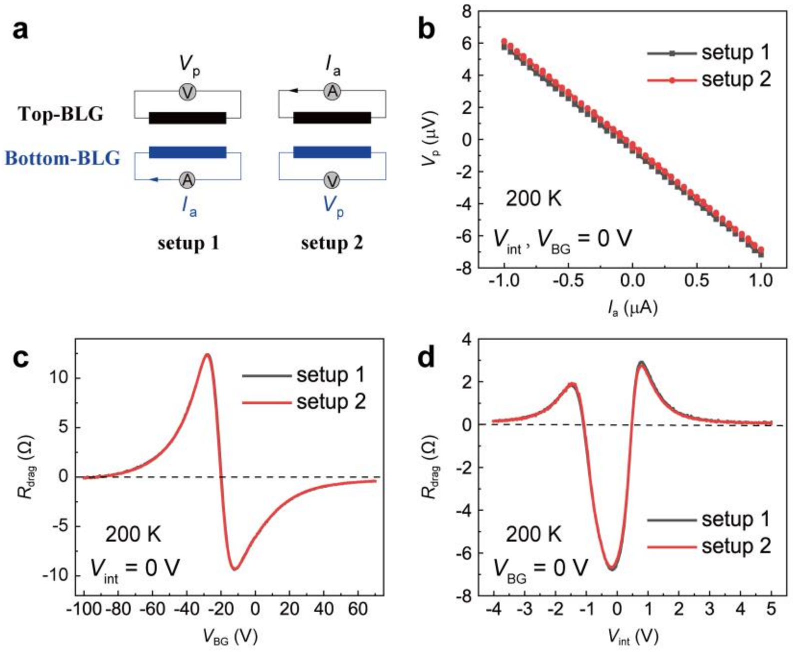

Supplementary Figure 2 | Verification of Onsager reciprocity relations. a, Schematic diagrams for two different setups of drag measurements. **b**, V_p vs I_a curves obtained using these two setups. V_{int} and V_{BG} are set to 0 during the measurements. **c**, R_{drag} vs V_{BG} (at V_{int} = 0 V) and **d**, R_{drag} vs V_{int} (at V_{BG} = 0 V) obtained using the two setups. All the measurements are conducted at T = 200 K.

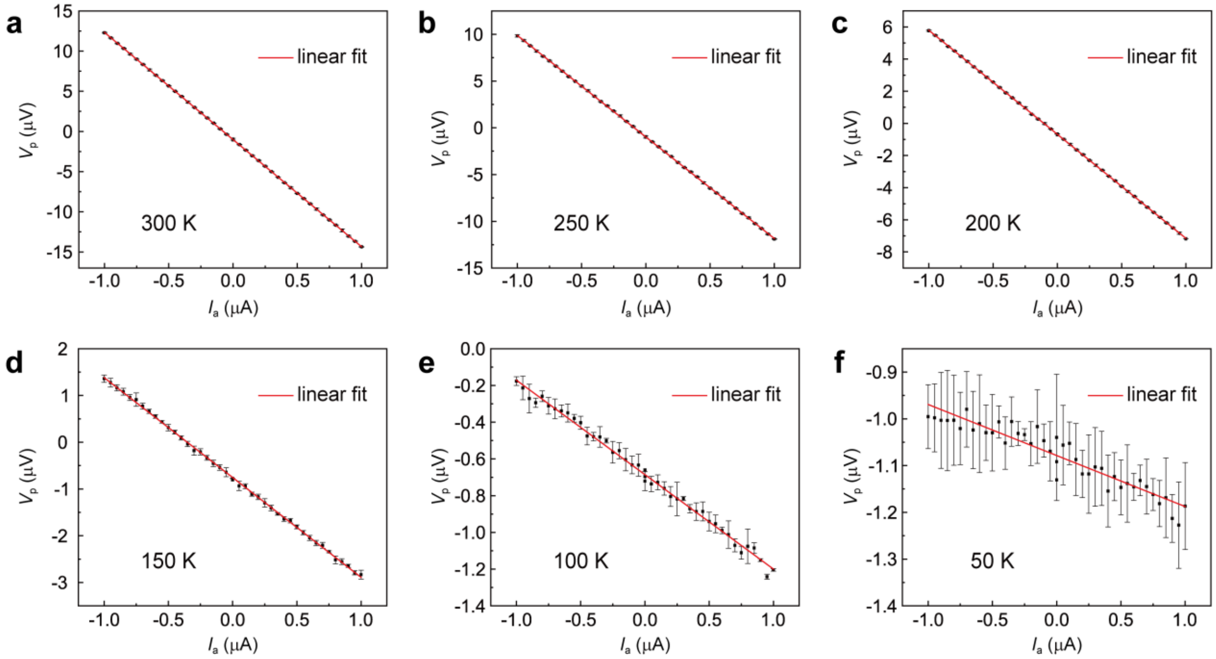

Supplementary Figure 3 | V_p - I_a characteristics at different temperatures. a-f, V_p - I_a curves obtained at different temperatures by using setup 1 (see Supplementary Fig. 2a). V_{int} and V_{BG} are set to 0 V during the measurements. The error bars represent the standard deviation from at least four measurements.

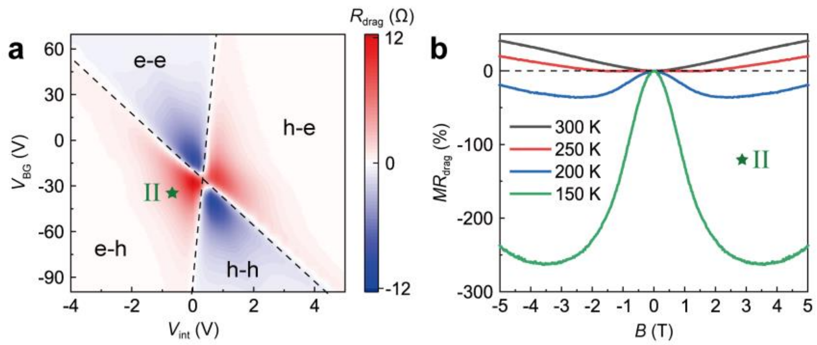

Supplementary Figure 4 | Evolution of magneto-drag behaviors with varying temperatures in the e-h region. **a**, R_{drag} vs (V_{int}, V_{BG}) at 200 K. **b**, MR_{drag} curves measured at different temperatures for point “II” indicated in **a**.

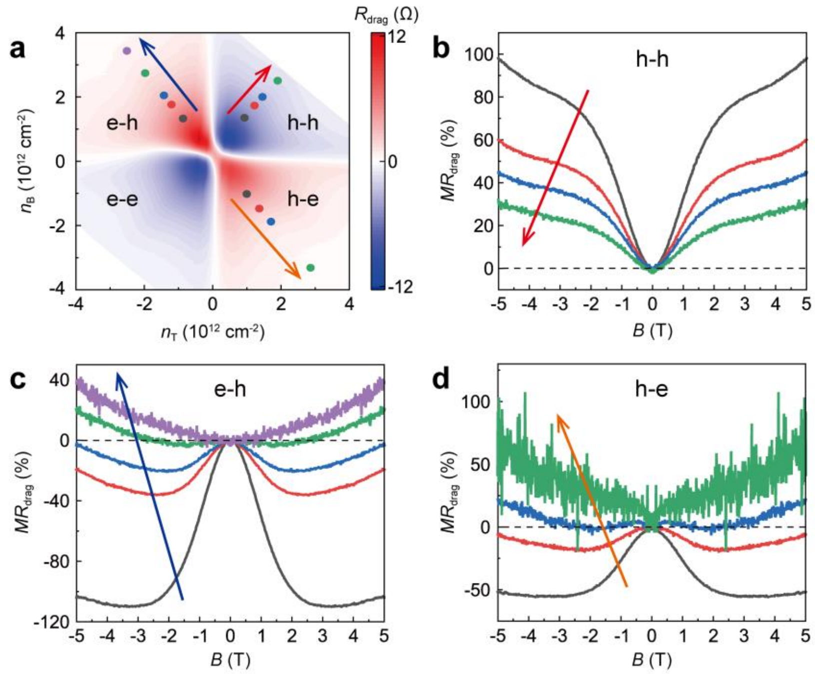

Supplementary Figure 5 | Evolution of magneto-drag behaviors with varying carrier densities. **a**, R_{drag} vs (n_T, n_B) at 200 K. **b-d**, MR_{drag} curves for typical points in the h-h, e-h and h-e regions, respectively (as indicated by the dots in **a**). All these measurements are conducted at T = 200 K.

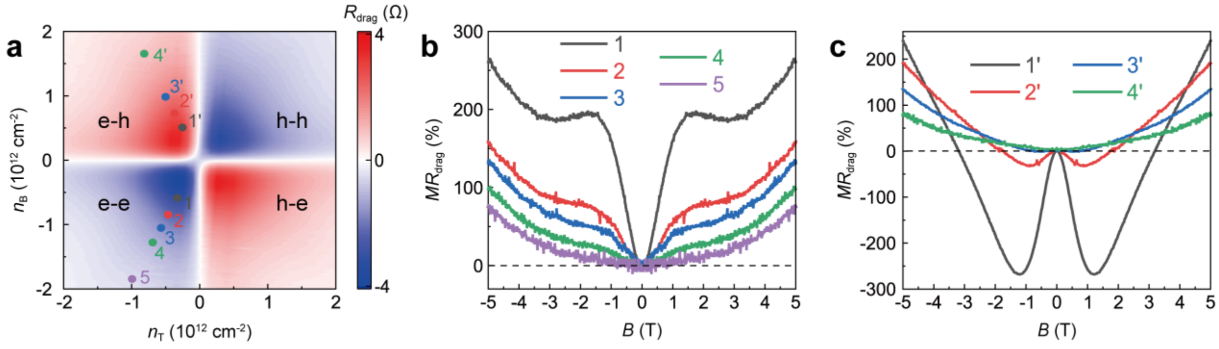

Supplementary Figure 6 | Magneto-drag behaviors in another bilayer/bilayer graphene (BLG/BLG) device. a, R_{drag} vs (n_T, n_B) in a BLG/BLG device with the thickness of hBN d~15.3 nm. **b**,**c**, MR_{drag} curves for typical points in the e-e region and e-h region, respectively (as indicated by the dots in **a**). All these measurements are conducted at T = 200 K.

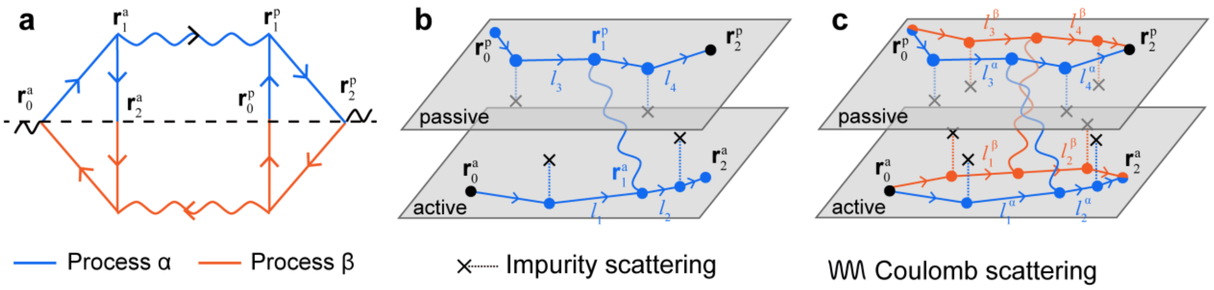

Supplementary Figure 7 | Schematics of drag process and the corresponding interference. a, One of the leading-order Feynman diagram for drag conductivity, where the two drag processes α and β are marked in blue and orange, respectively. **b**, Schematic of a typical drag process, describing how an active carrier at position $\mathbf{r}_0^{\mathrm{a}}$ induces a passive carrier moving towards $\mathbf{r}_2^{\mathrm{p}}$ (as detailed in Supplementary Note 4A). **c**, Interference between two drag processes, α (blue) and β (orange).

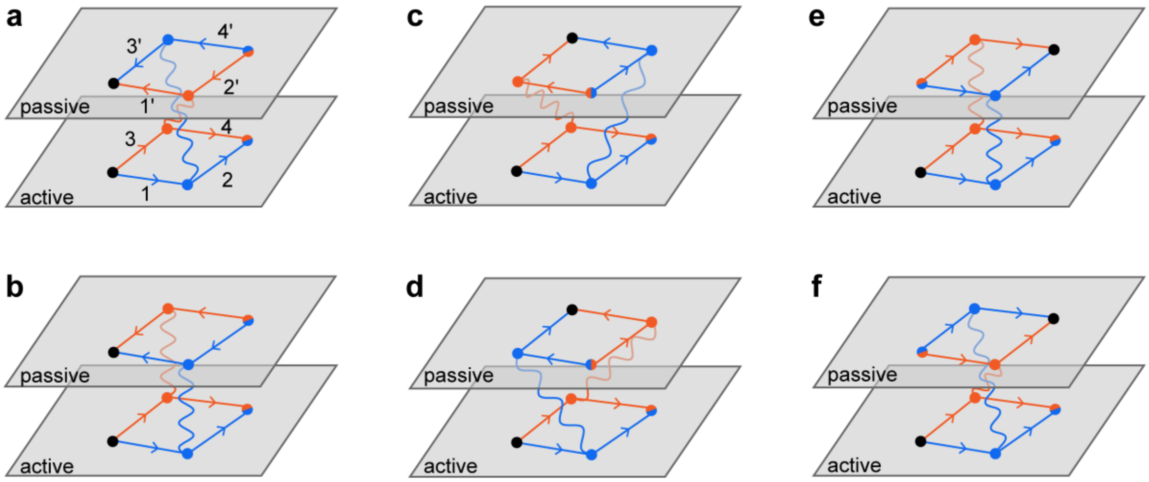

Supplementary Figure 8 | All possible scenarios of inter-layer quantum interference. a-f, Distinguishable types of interference containing four pairs of superimposing planar paths, labeled by (i, i') with $i = 1, 2, 3, 4$. The impurity scatterings of diffusion paths are not shown for simplicity.

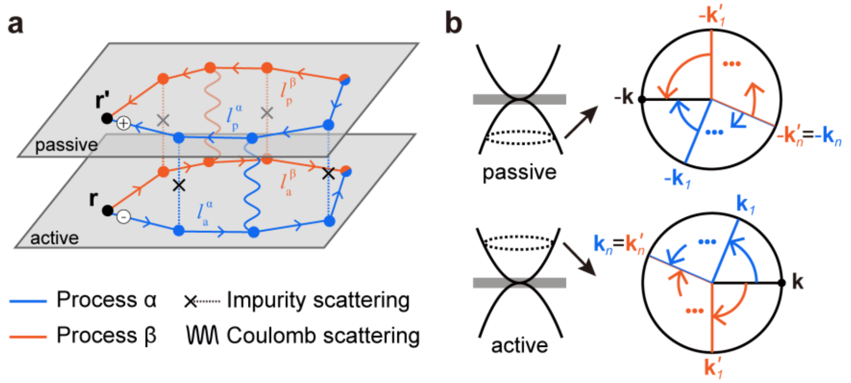

Supplementary Figure 9 | Inter-layer quantum interference (QI) in the e-h region. Schematics of inter-layer QI between two drag processes in the e-h region depicted in the **a**, real space and **b**, reciprocal space. Here, the paired superimposing planar paths (l_{a}^{α} and l_{p}^{α}, l_{a}^{β} and l_{p}^{β}) belong to the same drag process. This differs from the e-e case, in which the paired paths belong to different drag processes.

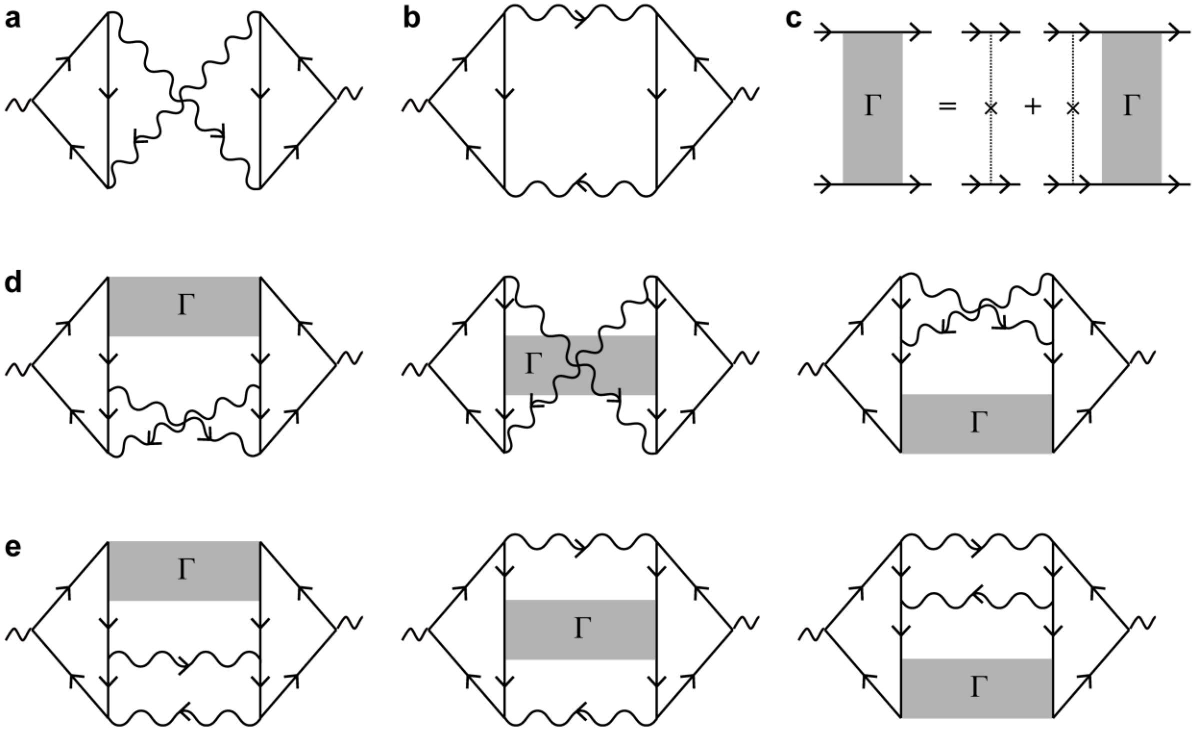

Supplementary Figure 10 | Feynman diagrams for inter-layer quantum interference (QI). **a**,**b**, Leading-order Feynman diagrams for drag conductivity. Here, **a** is topologically equivalent to Supplementary Fig. 7a. **c**, Inter-layer Cooperon ladder. The dotted lines with cross denote the impurity scatterings. **d**,**e**, Feynman diagrams for the correction arising from inter-layer QI in the **d**, e-e/h-h region and the **e**, e-h/h-e region, which are derived from Fig. 3b and Supplementary Fig. 9a respectively.

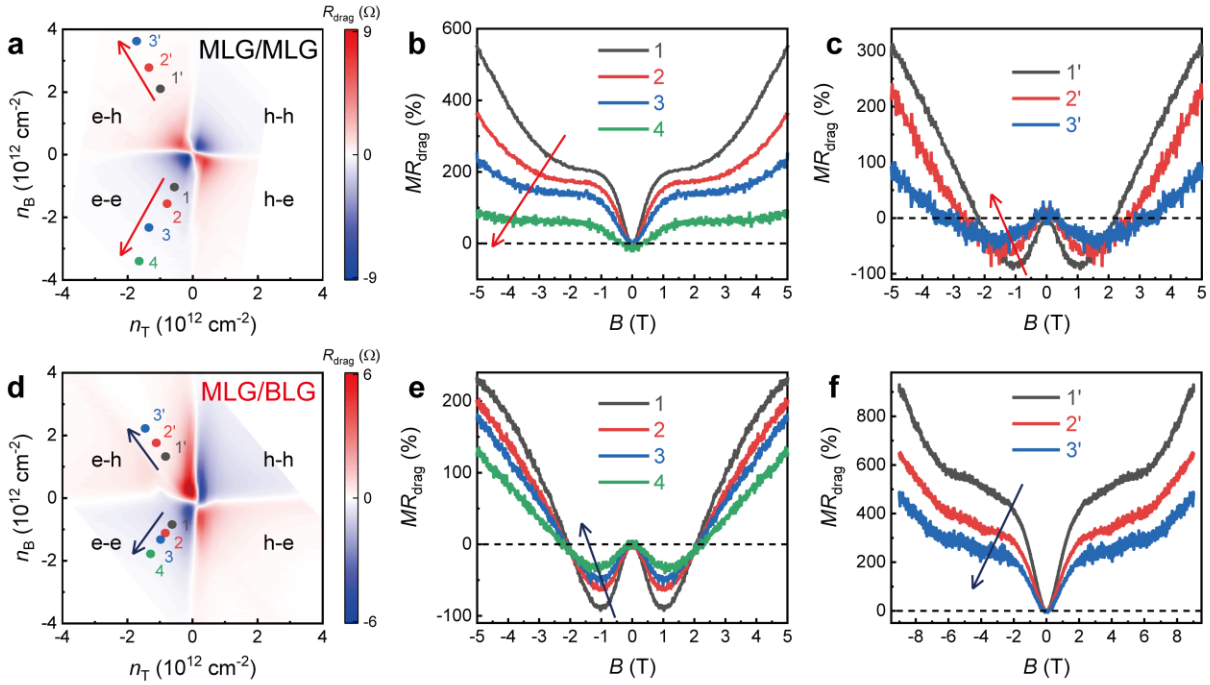

Supplementary Figure 11 | Evolution of magneto-drag behaviors with varying carrier densities in the monolayer/monolayer graphene (MLG/MLG) and monolayer/bilayer graphene (MLG/BLG) devices. a, R_{drag} vs (n_T, n_B) at 200 K for the MLG/MLG device. **b**,**c**, Corresponding MR_{drag} curves for typical points indicated in **a**. **d**, R_{drag} vs (n_T, n_B) at 200 K for the MLG/BLG device. **e**,**f**, Corresponding MR_{drag} curves for typical points indicated in **d**. All these measurements are conducted at T = 200 K. For both devices, the magnitude of low-field deviation of MR_{drag} becomes monotonically weaker as the carrier densities increase, identical to the ones observed in the BLG/BLG device (Fig. 2c and Supplementary Fig. 5).

Supplementary Table

Supplementary Table 1 | Phase analyses in the e-e/h-h region

Phase / Cases	φ_α (blue)	φ_β (orange)	$\varphi_\alpha - \varphi_\beta$
**A**	$\varphi_1 + \varphi_2 + \varphi_3 + \varphi_4$	$\varphi_1 + \varphi_2 + \varphi_3 + \varphi_4$	0
**B**	$2\varphi_1 + 2\varphi_2$	$2\varphi_3 + 2\varphi_4$	$2\varphi_1 + 2\varphi_2 - 2\varphi_3 - 2\varphi_4$
**C**	$\varphi_1 + 2\varphi_2 + \varphi_4$	$\varphi_1 + 2\varphi_3 + \varphi_4$	$2\varphi_2 - 2\varphi_3$
**D**	$2\varphi_1 + \varphi_2 + \varphi_3$	$\varphi_2 + \varphi_3 + 2\varphi_4$	$2\varphi_1 - 2\varphi_4$
**E**	$2\varphi_1 + 2\varphi_2$	$2\varphi_3 + 2\varphi_4$	$2\varphi_1 + 2\varphi_2 - 2\varphi_3 - 2\varphi_4$
**F**	$\varphi_1 + \varphi_2 + \varphi_3 + \varphi_4$	$\varphi_1 + \varphi_2 + \varphi_3 + \varphi_4$	0

note: Cases A-F are schematically shown in Supplementary Fig. 8.

Supplementary Table 2 | Phase analyses in the e-h/h-e region

Phase / Cases	φ_α (blue)	φ_β (orange)	$\varphi_\alpha - \varphi_\beta$
**A**	$\varphi_1 + \varphi_2 - \varphi_3 - \varphi_4$	$\varphi_3 + \varphi_4 - \varphi_1 - \varphi_2$	$2\varphi_1 + 2\varphi_2 - 2\varphi_3 - 2\varphi_4$
**B**	0	0	0
**C**	$\varphi_1 - \varphi_4$	$\varphi_4 - \varphi_1$	$2\varphi_1 - 2\varphi_4$
**D**	$\varphi_2 - \varphi_3$	$\varphi_3 - \varphi_2$	$2\varphi_2 - 2\varphi_3$
**E**	0	0	0
**F**	$\varphi_1 + \varphi_2 - \varphi_3 - \varphi_4$	$\varphi_3 + \varphi_4 - \varphi_1 - \varphi_2$	$2\varphi_1 + 2\varphi_2 - 2\varphi_3 - 2\varphi_4$

note: Cases A-F are schematically shown in Supplementary Fig. 8.

Supplementary Table 3 | Magnetic-field-induced phase difference in the e-e/h-h region

Phase / Cases	θ_α (blue)	θ_β (orange)	$\theta_\alpha - \theta_\beta$
**A**	$\theta_1 + \theta_2 - \theta_3 - \theta_4$	$\theta_3 + \theta_4 - \theta_1 - \theta_2$	$2\theta_1 + 2\theta_2 - 2\theta_3 - 2\theta_4$
**F**	$\theta_1 + \theta_2 + \theta_3 + \theta_4$	$\theta_1 + \theta_2 + \theta_3 + \theta_4$	0

note: Cases A and F are schematically shown in Supplementary Fig. 8.

Supplementary Table 4 | Magnetic-field-induced phase difference in the e-h/h-e region

Cases \ Phase	θ_α (blue)	θ_β (orange)	$\theta_\alpha - \theta_\beta$
**B**	$2\theta_1 + 2\theta_2$	$2\theta_3 + 2\theta_4$	$2\theta_1 + 2\theta_2 - 2\theta_3 - 2\theta_4$
**E**	0	0	0

note: Cases B and E are schematically shown in Supplementary Fig. 8.

Supplementary Note

Supplementary Note 1. Validity of drag measurements

To verify the validity of the drag measurements, we first conducted comparative experiments by interchanging the active and passive layers (two opposite setups shown in Supplementary Fig. 2a). As demonstrated in Supplementary Figs. 2b-d, the as-obtained drag curves using these two drag set-ups are nearly identical, well obeying the expected Onsager reciprocity relation.

We futher checked the V_p - I_a relationship at different temperatures using setup 1. A good liner V_p - I_a relation occurred at temperatures above 100 K (Supplementary Fig. 3). Lowering the temperature leads to unexpected fluctuations, hindering the extraction of accurate R_{drag} via linear fitting. Similar fluctuations have been observed previously, and were interpreted as mesoscopic drag fluctuations as a consequence of phase coherent quantum transport within the constituent layers [1-3].

Supplementary Note 2. Theoretical analysis on the high field B^2 dependence of drag resistance

The high field behavior of B^2 dependence for the drag resistance has been widely reported in previous experimental studies [4-6]. Below, we would like to give an explanation for such B^2 dependence based on the Drude-like model for Coulomb drag.

According to previous study [7], for a drag system wherein the two constituent graphene layers are identical, drag resistivity is given by

$$\rho_{\mathrm{xx}}^{\mathrm{D}} = \frac{r_0}{2}\left[\frac{1}{1-\left[1-\frac{n^2}{N^2}\right]\frac{R_{\mathrm{H}}^2}{R_{\mathrm{H}}^2+R_0^2}\bar{f}_+} - \frac{1}{1-\frac{n^2}{N^2}\frac{2R_0^{\mathrm{D}}}{2R_0^{\mathrm{D}}+r_0}-\left[1-\frac{n^2}{N^2}\right]\frac{R_{\mathrm{H}}^2}{R_{\mathrm{H}}^2+R_0^2+\frac{N^2-n^2}{n^2}2R_0^{\mathrm{D}}r_0}\bar{f}_-}\right] \quad (1)$$

Here $n = n_{\mathrm{e}} - n_{\mathrm{h}}$ and $N = n_{\mathrm{e}} + n_{\mathrm{h}}$ are the charge and quasiparticle densities in each layer, r_0 is the zero-field residual resistance of graphene at the charge neutral point, R_0 and R_{H} are the Drude

and Hall resistances far from the charge neutral point, R_0^{D} is the zero-field drag resistance far from the charge neutral point, and $\bar{f}_{\pm}$ is defined as

$$\bar{f}_{\pm} = 1 - \frac{\tanh(W/L_{\pm})}{W/L_{\pm}} \tag{2}$$

with

$$L_{+}^{-2} = \frac{e^2 n}{4\kappa\tau_{\mathrm{ph}}} R_0 \left[1 + \frac{R_{\mathrm{H}}^2}{R_0^2}\right], L_{-}^{-2} = \frac{e^2 n}{4\kappa} R_0 \left[\frac{1}{\tau_{\mathrm{ph}}} + \frac{1}{\tau_{\mathrm{Q}}}\right]\left[\frac{2R_0^{\mathrm{D}}}{r_0}\left(1 - \frac{n^2}{N^2}\right) + 1 + \frac{R_{\mathrm{H}}^2}{R_0^2}\right] \tag{3}$$

where W is sample width, κ is the mean quasiparticle kinetic energy, τ_{ph}^{-1} and τ_{Q}^{-1} are the quasiparticle relaxation rate due to the electron-phonon scattering and the inter-layer Coulomb interaction, respectively.

For a sample with small W ($W \ll L_{\pm}$), one will find $\bar{f}_{\pm} \approx W^2/(3L_{\pm}^2)$ and then we have

$$\rho_{\mathrm{xx}}^{\mathrm{D}} = \frac{r_0}{2}\left[\frac{1}{1 - \left[1 - \frac{n^2}{N^2}\right]\frac{(B\mu)^2}{1+(B\mu)^2}\bar{f}_{+}} - \frac{1}{1 - \frac{n^2}{N^2}\frac{2R_0^{\mathrm{D}}}{2R_0^{\mathrm{D}} + r_0} - \left[1 - \frac{n^2}{N^2}\right]\frac{(B\mu)^2}{1 + (B\mu)^2 + \left[1 - \frac{n^2}{N^2}\right]2R_0^{\mathrm{D}}/r_0}\bar{f}_{-}}\right]$$

$$= \frac{r_0}{2}\left[\left[1 - \frac{n^2}{N^2}\right]\frac{(B\mu)^2}{1+(B\mu)^2}\bar{f}_{+} - \frac{n^2}{N^2}\frac{2R_0^{\mathrm{D}}}{2R_0^{\mathrm{D}} + r_0} - \left[1 - \frac{n^2}{N^2}\right]\frac{(B\mu)^2}{1 + (B\mu)^2 + \left[1 - \frac{n^2}{N^2}\right]2R_0^{\mathrm{D}}/r_0}\bar{f}_{-}\right]$$

$$= -\frac{r_0}{2}\left[\frac{n^2}{N^2}\frac{2R_0^{\mathrm{D}}}{2R_0^{\mathrm{D}} + r_0} + \left[1 - \frac{n^2}{N^2}\right](B\mu W)^2\frac{e}{12\mu\kappa\tau_{\mathrm{Q}}}\right] \tag{4}$$

Here B is the magnetic field and μ is the carrier mobility. We can draw two conclusions from this expression: (i) The zero field drag resistivity, given by $-\frac{n^2}{N^2}\frac{R_0^{\mathrm{D}} r_0}{2R_0^{\mathrm{D}} + r_0}$, is negative. (ii) The magneto-drag resistivity follows a B^2 dependence. These results coincide with our experimental observations (Fig. 1e in the main text), indicating that the Drude-like model can well describe the classical magneto-drag behavior.

Supplementary Note 3. Repeatability of magneto-drag behaviors in BLG/BLG devices

Supplementary Fig. 4 presents the evolution of magneto-drag behaviors with varying temperatures in the e-h region. It is clearly seen that the low-field correction gets suppressed with increasing temperature (Supplementary Fig. 4b), consisting with the evolution behavior observed in the e-e region (see Fig. 2b in the main text).

In Supplementary Fig. 5 we further plotted the MR_{drag} data measured at different carrier densities for the h-h, e-h and h-e regions. All the MR_{drag} curves show a clear low-field deviation, with the magnitude monotonically weakening as the carrier densities of BLG layers increase. These observations also consist well with the results from the e-e region (Fig. 2c in the main text).

To further evaluate the repeatability of the magneto-drag behaviors, we conducted systematic measurements on another BLG/BLG device. Supplementary Fig. 6a shows R_{drag} as functions of n_{T} and n_{B} obtained at 200 K, from which four distinct regions are clearly seen. Low-field deviations are evident for the MR_{drag} curves taken in both the e-e region (Supplementary Fig. 6b) and the e-h region (Supplementary Fig. 6c), and show identical characteristics with those obtained in the device demonstrated in the main text (Fig. 2c in the main text and Supplementary Fig. 5c).

Supplementary Note 4. Theoretical analysis of quantum interference between inter-layer drag processes

A. Probability amplitude of the drag process

According to the leading-order Feynman diagram of drag conductivity (Supplementary Fig. 7a) [8, 9], a typical drag process can be described as follows (Supplementary Fig. 7b): 1) A carrier in the active layer ("active carrier") starts at $\mathbf{r}_0^{\mathrm{a}}$ and a carrier in the passive layer ("passive carrier") at $\mathbf{r}_0^{\mathrm{p}}$; 2) These two carriers interact Coulombically when they travel separately to $\mathbf{r}_1^{\mathrm{a}}$ and $\mathbf{r}_1^{\mathrm{p}}$ along the diffusion paths l_1 and l_3; 3) After the Coulomb scattering, they end up at $\mathbf{r}_2^{\mathrm{a}}$ and $\mathbf{r}_2^{\mathrm{p}}$ after traveling along paths l_2 and l_4, respectively. The probability amplitude ψ of such an inter-layer transport process is given by:

$$\psi = G_{l_4}(\mathbf{r}_2^{\mathrm{p}}, \mathbf{r}_1^{\mathrm{p}}) G_{l_2}(\mathbf{r}_2^{\mathrm{a}}, \mathbf{r}_1^{\mathrm{a}}) V(\mathbf{r}_1^{\mathrm{p}}, \mathbf{r}_1^{\mathrm{a}}) G_{l_3}(\mathbf{r}_1^{\mathrm{p}}, \mathbf{r}_0^{\mathrm{p}}) G_{l_1}(\mathbf{r}_1^{\mathrm{a}}, \mathbf{r}_0^{\mathrm{a}}) \quad (5)$$

Here, V describes the inter-layer Coulomb interaction and G_l is the propagation amplitude along diffusion path l. We note that the inter-layer Coulomb interaction will transfer energy $\Delta\varepsilon$ from the active layer to the passive layer, and thus introduce phase factors $e^{\mathrm{i}\Delta\varepsilon t}$ and $e^{-\mathrm{i}\Delta\varepsilon t}$ to the

propagating amplitudes of the active carrier and the passive carrier, respectively. However, these two phase factors are involved in G_{l_2} and G_{l_4}, respectively, and will cancel out with each other eventually. That is, the inter-layer Coulomb interaction does not lead to any dephasing for the overall drag process.

Then the probability $P(\mathbf{r}_2^{\mathrm{p}}, \mathbf{r}_0^{\mathrm{a}})$ for an active carrier at $\mathbf{r}_0^{\mathrm{a}}$ inducing a passive carrier moving to $\mathbf{r}_2^{\mathrm{p}}$ is:

$$P(\mathbf{r}_2^{\mathrm{p}}, \mathbf{r}_0^{\mathrm{a}}) = \int d\mathbf{r}_0^{\mathrm{p}} d\mathbf{r}_2^{\mathrm{a}} \left| \int d\mathbf{r}_1^{\mathrm{a}} d\mathbf{r}_1^{\mathrm{p}} \sum_{l_1 l_2 l_3 l_4} \psi \right|^2 \tag{6}$$

Here, $\mathbf{r}_2^{\mathrm{a}}$ and $\mathbf{r}_0^{\mathrm{p}}$ are integrated since they are not recorded in the drag measurement. As the drag conductivity can be obtained from $P(\mathbf{r}_2^{\mathrm{p}}, \mathbf{r}_0^{\mathrm{a}})$ and the knowledge of current vertices in Supplementary Fig. 7a, analysis of $P(\mathbf{r}_2^{\mathrm{p}}, \mathbf{r}_0^{\mathrm{a}})$ can provide a qualitative understanding of the drag conductivity/current.

B. Inter-layer interference between drag processes

For a pair of drag processes "α" and "β" (see Supplementary Fig. 7c), the interference term in $P(\mathbf{r}_2^{\mathrm{p}}, \mathbf{r}_0^{\mathrm{a}})$ is given by $2|\psi_\alpha \psi_\beta| \cos(\varphi_\alpha - \varphi_\beta)$. Here, ψ and φ represent the probability amplitude of the drag process and the corresponding phase, respectively. Note that these two drag processes α and β have the same initial and final states, i.e., $\{\mathbf{r}_0^{\mathrm{a}}, \mathbf{r}_0^{\mathrm{p}}\}$ and $\{\mathbf{r}_2^{\mathrm{a}}, \mathbf{r}_2^{\mathrm{p}}\}$, which is necessary for the emergence of interference. As a result, the diffusion paths involved in the interference, i.e., $\{l_1^\alpha, l_2^\alpha, l_1^\beta, l_2^\beta\}$ and $\{l_3^\alpha, l_4^\alpha, l_3^\beta, l_4^\beta\}$, form a closed loop within both the active and passive layers (Supplementary Fig. 7c). The phase of the probability amplitude for drag process α or β can be obtained by adding up the phases of constituent diffusion paths:

$$\varphi_{\alpha/\beta} = \sum_{i=1,2,3,4} \varphi_{l_i^{\alpha/\beta}} \tag{7}$$

In general, interferences of nearly all pairs of drag processes cancel out after being averaged over all possible diffusion paths. Only pairs of drag processes with constant $\varphi_\alpha - \varphi_\beta$ that are independent of constituent paths will have observable interference effect that contributes to the

drag signal. To identify these paired drag processes, we first divide the corresponding eight diffusion paths, i.e., $\{l_i^\alpha, l_i^\beta \mid i = 1, 2, 3, 4\}$, into four groups of two. The two paths in each group are required to have a definite phase relation and contribute to a constant in $\varphi_\alpha - \varphi_\beta$. Here, a definite phase relation refers to either a constant difference or a sign reversal, depending on that these two paths belong to different drag processes or the same one. For inter-layer QI, these two paths are further required to reside respectively in the two separate layers.

The above requirements can be satisfied only when the impurity potentials acting on the two layers are identical, and thus carriers from different layers could maintain their motions along superimposing planar paths. Here, a pair of superimposing planar paths are interrelated by either inter-layer mirror reflection or a combination of inter-layer mirror reflection and time reversal. Supplementary Fig. 8 shows all the possible scenarios of interference containing four pairs of superimposing planar paths, labeled by (i, i') with $i = 1, 2, 3, 4$. Below, we present systematic phase analyses for these scenarios, which were performed separately for the e-e/h-h and e-h/h-e regions.

(1) e-e/h-h region

For the e-e/h-h region, the propagation amplitudes along the two superimposing planar paths have the same phase, that is:

$$\varphi_i = \varphi_{i'} \ (i = 1, 2, 3, 4). \tag{8}$$

Here, we note that time reversal does not change the propagation amplitude. Supplementary Table 1 lists the calculated phase difference of two drag processes $\varphi_\alpha - \varphi_\beta$, from which only the scenarios shown in Supplementary Figs. 8a,f can survive the impurity average since the overall phase differences for these circumstances are constant and therefore independent of constituent diffusion paths.

(2) e-h/h-e region

For the e-h/h-e region, the propagation amplitudes along the two superimposing planar paths have opposite phases (see Supplementary Note 4E), that is:

$$\varphi_i = -\varphi_{i'} \ (i = 1, 2, 3, 4). \tag{9}$$

After conducting analyses similar to the e-e/h-h case, we conclude that only the scenarios shown in Supplementary Figs. 8b,e survive the impurity average (Supplementary Table 2).

C. Magnetic-field-induced phase difference

When a vertical magnetic field is applied, an additional phase ($\theta = \pm\int_{c}\mathbf{A}\cdot dl$) arises for a carrier propagating along path l under a vector potential $\mathbf{A}$, with its sign depending on the polarity and the propagation direction of the carrier. For the above identified pairs of drag processes, the corresponding θ can be readily obtained, as listed in Supplementary Table 3 and Table 4. It is concluded that only the interference shown in Supplementary Fig. 8a (Fig. 8b) for the e-e/h-h region (e-h/h-e region) is destroyed by the magnetic field, and responsible for the observed low-field corrections of drag resistance.

D. Quantum correction to the drag resistance

The finally established inter-layer QI for the e-e and e-h regions are schematically shown in Fig. 3b (right panel) in the main text and Supplementary Fig. 9a, respectively. Each pair of superimposing planar paths in these interferences are interrelated by inter-layer mirror reflection and time reversal. For convenience, we define $l_{\mathrm{a}}^{\alpha} = \{l_1^{\alpha}, l_2^{\alpha}\}$ and $l_{\mathrm{p}}^{\alpha} = \{l_3^{\alpha}, l_4^{\alpha}\}$. As detailed in the main text, in the reciprocal space, the drag process involved in the inter-layer QI corresponds to the case that an active carrier with momentum $\mathbf{k}$ scatters a passive carrier into the $-\mathbf{k}$ state (Fig. 3c in the main text and Supplementary Fig. 9b), which can be referred to as inter-layer backscattering.

Assuming that the drive current is $I_{\mathrm{a}} = q_{\mathrm{a}}v_{\mathrm{a}}(\mathbf{k})$, the correction induced by the above inter-layer QI to the drag current would be $I_{\mathrm{p}}^{\mathrm{cor}} = q_{\mathrm{p}}v_{\mathrm{p}}(-\mathbf{k})$, with its direction opposite to I_{a} for both the e-e and e-h regions. Here, q and v are the charge and velocity of carrier respectively. We note that in our model, a hole with momentum $\mathbf{k}$ means that the $\mathbf{k}$ state is unoccupied. Thus, the hole with momentum $-\mathbf{k}$ in the conduction band has the same velocity as the electron with momentum $\mathbf{k}$ in valence band.

Similar to the analysis for the e-e region presented in the main text, here we discuss the correction of inter-layer QI to the drag resistance in the e-h region. For typical Coulomb drag obeying momentum transfer mechanism, the classical drag current $I_{\mathrm{p}}^{\mathrm{cla}}$ will flow along the opposite direction as the drive current I_{a} in the e-h region. The enhancement of inter-layer backscattering

due to QI will leads to an increase in the total drag current (I_p^{cla} + I_p^{cor}). The corresponding increase in accumulated open-circuit voltage between the electrodes (i.e., the measured V_p) leads to an increase in the magnitude of drag resistance. This is consistent with the experimentally obtained deviation of zero-field R_{drag} (Fig. 1f in the main text).

As detailed in Supplementary Note 4C, applying a magnetic field will break the interference. Thus, the quantum correction to the drag current I_p^{cor} will be suppressed with increasing magnetic field, leading to negative MR_{drag} for the e-h region at low-field regime. This conclusion also consists well with our experimental data (Supplementary Figs. 4b and 5c).

E. Propagation amplitudes of carriers

To investigate the impurity scatterings of a carrier in a crystal, the T-matrix in scattering theory is defined as:

$$T = V^{\mathrm{I}} + V^{\mathrm{I}}\frac{1}{\varepsilon - H_0}V^{\mathrm{I}} + V^{\mathrm{I}}\frac{1}{\varepsilon - H_0}V^{\mathrm{I}}\frac{1}{\varepsilon - H_0}V^{\mathrm{I}}\cdots \tag{10}$$

Here, V^{I} is the impurity potential, H_0 is the Hamiltonian of the perfect crystal, and ε is the energy of carrier.

For convenience, we will discuss the propagation amplitude in the reciprocal (quasi-momentum) space, and the conclusions hold for the real space as well. A diffusion path in the reciprocal space can be defined as a sequence:

$$l = \{\mathbf{k}, \mathbf{k}_1, \cdots, \mathbf{k}_n, \mathbf{k}'\} \tag{11}$$

where $\mathbf{k}$ and $\mathbf{k}'$ are the initial and final momenta, respectively, and $\mathbf{k}_i$ are intermediate momenta.

For monolayer and bilayer graphene, we will consider the commonly used low-energy two band models [10-12] with band index $s = \pm 1$ corresponding to conduction and valence bands, respectively. In these models, the matrix elements of the impurity potential are independent of band index: $\langle s\mathbf{k}'|V^{\mathrm{I}}|s\mathbf{k}\rangle = \langle -s\mathbf{k}'|V^{\mathrm{I}}|-s\mathbf{k}\rangle = V^{\mathrm{I}}_{\mathbf{k}'\mathbf{k}}$ [11]. For an electron in the conduction band with $s = +1$, the propagation amplitude along l is:

$$T^e = V^{\mathrm{I}}_{\mathbf{k}'\mathbf{k}_n}\frac{1}{\varepsilon - \varepsilon^e_{+1,\mathbf{k}_n}}\cdots V^{\mathrm{I}}_{\mathbf{k}_2\mathbf{k}_1}\frac{1}{\varepsilon - \varepsilon^e_{+1,\mathbf{k}_1}}V^{\mathrm{I}}_{\mathbf{k}_1\mathbf{k}} \tag{12}$$

In this expression, inter-band transitions are ignored.

For a hole in the valence band with $s=-1$, the propagation amplitude along l is:

$$T^h = V^{\mathrm{I}}_{\mathbf{k}_n\mathbf{k}'} \frac{1}{\varepsilon - \varepsilon^h_{-1,\mathbf{k}_n}} \cdots V^{\mathrm{I}}_{\mathbf{k}_1\mathbf{k}_2} \frac{1}{\varepsilon - \varepsilon^h_{-1,\mathbf{k}_1}} V^{\mathrm{I}}_{\mathbf{k}\mathbf{k}_1} \tag{13}$$

We note that here, a hole with momentum **k** means that the **k** state is unoccupied. The energy of hole is $\varepsilon^h_{-1,\mathbf{k}} = -\varepsilon^e_{-1,\mathbf{k}} = \varepsilon^e_{+1,\mathbf{k}}$. We then find that the probability amplitudes of an electron in the conduction band and a hole in the valence band propagating along the same diffusion path are complex conjugate, i.e., $T^e = (T^h)^*$.

Supplementary Note 5. Mathematical analysis of the drag resistivity

A. Classical drag resistivity

Given the conductivity matrix $\boldsymbol{\sigma} = \begin{pmatrix} \sigma_1 & \sigma_\mathrm{d} \\ \sigma_\mathrm{d} & \sigma_2 \end{pmatrix}$, where σ_1 and σ_2 are the classical conductivity of each layer respectively, and σ_d is the classical drag conductivity, the resistivity matrix is [8, 13]:

$$\boldsymbol{\rho} = \begin{pmatrix} \rho_1 & \rho_\mathrm{d} \\ \rho_\mathrm{d} & \rho_2 \end{pmatrix} = \boldsymbol{\sigma}^{-1} = \begin{pmatrix} \frac{\sigma_2}{\sigma_1\sigma_2 - \sigma_\mathrm{d}^2} & \frac{-\sigma_\mathrm{d}}{\sigma_1\sigma_2 - \sigma_\mathrm{d}^2} \\ \frac{-\sigma_\mathrm{d}}{\sigma_1\sigma_2 - \sigma_\mathrm{d}^2} & \frac{\sigma_1}{\sigma_1\sigma_2 - \sigma_\mathrm{d}^2} \end{pmatrix} \approx \begin{pmatrix} \frac{1}{\sigma_1} & \frac{-\sigma_\mathrm{d}}{\sigma_1\sigma_2} \\ \frac{-\sigma_\mathrm{d}}{\sigma_1\sigma_2} & \frac{1}{\sigma_2} \end{pmatrix} \tag{14}$$

Here, $\sigma_1, \sigma_2 \gg \sigma_\mathrm{d}$. The classical drag resistivity is $\rho_\mathrm{d} = \frac{-\sigma_\mathrm{d}}{\sigma_1\sigma_2}$, which is a direct measurement of the inter-layer Coulomb interaction [14].

B. Intra-layer quantum correction

Taking the conventional intra-layer QI effect into account, the conductivities of the active and passive layers are $\sigma_1(1+\eta_1)$ and $\sigma_2(1+\eta_2)$, respectively, where $\sigma_1\eta_1$ and $\sigma_2\eta_2$ correspond to the quantum corrections in each layer. According to previous theoretical studies [8, 9], the drag conductivity is $\sigma_\mathrm{d}(1+\eta_1+\eta_2)$. The whole conductivity matrix is therefore:

$$\boldsymbol{\sigma} = \begin{pmatrix} \sigma_1(1+\eta_1) & \sigma_\mathrm{d}(1+\eta_1+\eta_2) \\ \sigma_\mathrm{d}(1+\eta_1+\eta_2) & \sigma_2(1+\eta_2) \end{pmatrix} \tag{15}$$

Since $\sigma_1, \sigma_2 \gg \sigma_\mathrm{d}$ and $\eta_1, \eta_2 \ll 1$, the resistivity matrix $\boldsymbol{\rho}$ up to the first order of η_1 and η_2 is:

$$\boldsymbol{\rho} = \boldsymbol{\sigma}^{-1} \approx \begin{pmatrix} \dfrac{(1-\eta_1)}{\sigma_1} & \dfrac{-\sigma_\mathrm{d}}{\sigma_1\sigma_2} \\ \dfrac{-\sigma_\mathrm{d}}{\sigma_1\sigma_2} & \dfrac{(1-\eta_2)}{\sigma_2} \end{pmatrix} \tag{16}$$

As such, the intra-layer quantum correction to the magneto-drag resistance is negligible [8].

C. Inter-layer quantum correction

For the inter-layer QI effect, the conductivity matrix can be rewritten as:

$$\boldsymbol{\sigma} = \begin{pmatrix} \sigma_1(1+\eta_1) & \sigma_\mathrm{d}(1+\eta_1+\eta_2)+\sigma_\mathrm{d}^\mathrm{cor} \\ \sigma_\mathrm{d}(1+\eta_1+\eta_2)+\sigma_\mathrm{d}^\mathrm{cor} & \sigma_2(1+\eta_2) \end{pmatrix} \tag{17}$$

Here, $\sigma_\mathrm{d}^\mathrm{cor}$ is the inter-layer-QI-induced correction to the drag conductivity. The resistivity $\boldsymbol{\rho}$ up to the first order of QI effect is therefore:

$$\boldsymbol{\rho} = \boldsymbol{\sigma}^{-\mathbf{1}} \approx \begin{pmatrix} \dfrac{(1-\eta_1)}{\sigma_1} & -\dfrac{\sigma_\mathrm{d}+\sigma_\mathrm{d}^\mathrm{cor}}{\sigma_1\sigma_2} \\ -\dfrac{\sigma_\mathrm{d}+\sigma_\mathrm{d}^\mathrm{cor}}{\sigma_1\sigma_2} & \dfrac{(1-\eta_2)}{\sigma_2} \end{pmatrix} \tag{18}$$

In addition to the classical drag resistivity $\rho_\mathrm{d}^\mathrm{cla} = \frac{-\sigma_\mathrm{d}}{\sigma_1\sigma_2}$, an additional inter-layer quantum correction to the drag resistivity is given as $\rho_\mathrm{d}^\mathrm{cor} = \frac{-\sigma_\mathrm{d}^\mathrm{cor}}{\sigma_1\sigma_2}$.

Supplementary References

[1] Price, A., Savchenko, A., Narozhny, B., Allison, G. & Ritchie, D. Giant fluctuations of coulomb drag in a bilayer system. *Science* **316**, 99-102 (2007).

[2] Gamucci, A. *et al.* Anomalous low-temperature Coulomb drag in graphene-GaAs heterostructures. *Nat. Commun.* **5**, 1-7 (2014).

[3] Zhu, L. *et al.* Frictional Drag Effect between Massless and Massive Fermions in Single-Layer/Bilayer Graphene Heterostructures. *Nano lett.* **20**, 1396-1402 (2020).

[4] Hill, N. *et al.* Frictional drag between parallel two-dimensional electron gases in a perpendicular magnetic field. *J. Phys.: Condens. Matter* **8**, L557 (1996).

[5] Gorbachev, R. *et al.* Strong Coulomb drag and broken symmetry in double-layer graphene. *Nat. Phys.* **8**, 896-901 (2012).

[6] Liu, X. *et al.* Frictional magneto-Coulomb drag in graphene double-layer heterostructures. *Phys. Rev. Lett.* **119**, 056802 (2017).

[7] Titov, M. *et al.* Giant magnetodrag in graphene at charge neutrality. *Phys. Rev. Lett.* **111**, 166601 (2013).

[8] Flensberg, K., Hu, B. Y.-K., Jauho, A.-P. & Kinaret, J. M. Linear-response theory of Coulomb drag in coupled electron systems. *Phys. Rev. B* **52**, 14761 (1995).

[9] Kamenev, A. & Oreg, Y. Coulomb drag in normal metals and superconductors: Diagrammatic approach. *Phys. Rev. B* **52**, 7516 (1995).

[10] Wallace, P. R. The band theory of graphite. *Phys. Rev.* **71**, 622 (1947).

[11] Ando, T., Nakanishi, T. & Saito, R. Berry's phase and absence of back scattering in carbon nanotubes. *J. Phys. Soc. Jpn.* **67**, 2857-2862 (1998).

[12] McCann, E. & Fal’ko, V. I. Landau-level degeneracy and quantum Hall effect in a graphite bilayer. *Phys. Rev. Lett.* **96**, 086805 (2006).

[13] Lux, J. & Fritz, L. Kinetic theory of Coulomb drag in two monolayers of graphene: from the Dirac point to the Fermi liquid regime. *Phys. Rev. B* **86**, 165446 (2012).

[14] Narozhny, B. & Levchenko, A. Coulomb drag. *Rev. Mod. Phys.* **88**, 025003 (2016).
